# Utilization of Cervical Cancer Screening Services and Trends in Screening Positivity Rates in a ‘Screen-And-Treat’ Program Integrated with HIV/AIDS Care in Zambia

**DOI:** 10.1371/journal.pone.0074607

**Published:** 2013-09-18

**Authors:** Mulindi H. Mwanahamuntu, Vikrant V. Sahasrabuddhe, Meridith Blevins, Sharon Kapambwe, Bryan E. Shepherd, Carla Chibwesha, Krista S. Pfaendler, Gracilia Mkumba, Belington Vwalika, Michael L. Hicks, Sten H. Vermund, Jeffrey SA. Stringer, Groesbeck P. Parham

**Affiliations:** 1 Center for Infectious Disease Research in Zambia, Lusaka, Zambia; 2 University Teaching Hospital, Lusaka, Zambia; 3 Vanderbilt University, Nashville, Tennessee, United States of America; 4 University of North Carolina at Chapel Hill, Chapel Hill, North Carolina, United States of America; 5 University of Cincinnati, Cincinnati, Ohio, United States of America; 6 Michigan Cancer Institute, Pontiac, Michigan, United States of America; University of Missouri Kansas CIty School of Medicine, United States of America

## Abstract

**Background:**

In the absence of stand-alone infrastructures for delivering cervical cancer screening services, efforts are underway in sub-Saharan Africa to dovetail screening with ongoing vertical health initiatives like HIV/AIDS care programs. Yet, evidence demonstrating the utilization of cervical cancer prevention services in such integrated programs by women of the general population is lacking.

**Methods:**

We analyzed program operations data from the Cervical Cancer Prevention Program in Zambia (CCPPZ), the largest public sector programs of its kind in sub-Saharan Africa. We evaluated patterns of utilization of screening services by HIV serostatus, examined contemporaneous trends in screening outcomes, and used multivariable modeling to identify factors associated with screening test positivity.

**Results:**

Between January 2006 and April 2011, CCPPZ services were utilized by 56,247 women who underwent cervical cancer screening with visual inspection with acetic acid (VIA), aided by digital cervicography. The proportion of women accessing these services who were HIV-seropositive declined from 54% to 23% between 2006–2010, which coincided with increasing proportions of HIV-seronegative women (from 22% to 38%) and women whose HIV serostatus was unknown (from 24% to 39%) (all p-for trend<0.001). The rates of VIA screening positivity declined from 47% to 17% during the same period (p-for trend <0.001), and this decline was consistent across all HIV serostatus categories. After adjusting for demographic and sexual/reproductive factors, HIV-seropositive women were more than twice as likely (Odds ratio 2.62, 95% CI 2.49, 2.76) to screen VIA-positive than HIV-seronegative women.

**Conclusions:**

This is the first ‘real world’ demonstration in a public sector implementation program in a sub-Saharan African setting that with successful program scale-up efforts, nurse-led cervical cancer screening programs targeting women with HIV can expand and serve all women, regardless of HIV serostatus. Screening program performance can improve with adequate emphasis on training, quality control, and telemedicine-support for nurse-providers in clinical decision making.

## Introduction

Invasive cervical cancer (ICC) is a leading cause of cancer-related mortality and morbidity among women in the developing world [1], [2]. Simplified “screen and treat” approaches [such as visual inspection with acetic acid (VIA) and immediate cryotherapy] for secondary prevention of cervical cancer have been developed for field adoption in resource-constrained settings where implementation and expansion of cytology (Pap smear)-based screening programs have proven unsustainable and human papillomavirus (HPV) testing is not yet available [3]–[8].

Stand-alone infrastructures for delivering public sector-based cervical cancer screening services are largely unavailable or poorly developed in most low and middle income countries [2]. In response, several international efforts are currently underway in sub-Saharan Africa to dovetail cervical cancer screening with ongoing vertical health initiatives, prominently with HIV/AIDS care and treatment programs [8]–[10]. In general, HIV-seropositive women are at increased risk of HPV persistence and cervical cancer incidence. Access to affordable combination antiretroviral therapy (cART) over the past decade has led to longer lifespans among HIV-seropositive women. Yet, they continue to be at higher risk for cervical cancer given lack of access to cervical cancer screening [11], [12]. Thus, provision of cervical cancer screening is a clinical care imperative for HIV-seropositive women. Several efforts have been undertaken in the past decade to develop cervical cancer screening infrastructures linked to HIV/AIDS care and treatment programs and targeting HIV-seropositive women, but open for access to all women, regardless of HIV serostatus [8]–[10], [13]–[17]. Nonetheless, evidence demonstrating the utilization and uptake of cervical cancer services offered in this format by women of the general population, beyond those who are HIV-seropositive, is lacking.

Zambia, a sub-Saharan African nation with a population over 13 million, has the world’s second highest annual cervical cancer incidence and mortality rates [1], as well as a generalized HIV/AIDS epidemic [18]. In 2006, the Cervical Cancer Prevention Program in Zambia (CCPPZ) was initiated as an innovative public sector service platform, built within and tightly integrated to U.S. President’s Emergency Plan for AIDS Relief (PEPFAR)-supported HIV/AIDS care and treatment infrastructures [10]. Notwithstanding the physical co-location of the CCPPZ clinics with HIV/AIDS clinics, cervical cancer screening services are open to all women, regardless of their HIV serostatus. In CCPPZ clinics, trained nurses provide ‘screen-and-treat’ services on the same day, or refer cryotherapy-ineligible women for further diagnostic evaluation and treatment to an outpatient surgery clinic located in a tertiary hospital. Over the course of seven years (2006 to present: June 2013), CCPPZ has provided screening services to over 100,000 women, and is the largest of such programs in sub-Saharan Africa.

We sought to evaluate the utilization of screening services by Zambian women stratified by HIV serostatus and examine the trends and factors associated with screening-test positivity over the program scale-up period, with a goal to inform the roll-out of similar programs being implemented in other resource constrained settings.

## Methods

The operational details of CCPPZ have been reported in previous manuscripts [10], [19]–[21]. Briefly, trained female Zambian nurses perform screening of women with VIA aided by digital camera enhancement of the cervix (digital cervicography). A same-day treatment by cryotherapy is offered to eligible VIA-positive women, and those ineligible for cryotherapy are referred to an outpatient Gynecologic Cancer Prevention Clinic for histopathological evaluation by biopsy or loop electrosurgical excision procedure (LEEP). Participant clinical and demographic information is entered electronically, at the point of care, into a database by community health workers and nurses that is updated weekly. Women attending CCPPZ clinics are asked to self-report HIV-status, and HIV- negative or indeterminate status women who agree to be tested are provided an on-site HIV test by the CCPPZ nurses. Thus, although HIV serostatus confirmation is by self-report, most HIV-seropositive women are ‘linked’ to the HIV/AIDS care and treatment clinics co-located and operational in the same premises.

For this study, for evaluating programmatic outcomes we analyzed de-identified patient records and operations data utilizing unique (baseline, not follow-up) visits of women accessing screening. We focused on outcomes and trends of two key program indicators: proportion of women who were HIV-seropositive and proportion of women who were VIA-positive. We compared clinical and demographic characteristics by HIV serostatus utilizing Chi-square statistic for categorical variables and the non-parametric Wilcoxon rank sum test for continuous or ordinal variables [22]. We used logistic regression to identify factors independently associated with VIA positivity, adjusting for key clinical and demographic predictors (Age, education, marital status, occupation, family income, number of lifetime sexual partners, age of sexual debut, number of pregnancies, condom use with regular partner, and HIV serostatus) that were selected *apriori.* Missing values of predictors were multiply imputed to prevent case-wise deletion [23]. To estimate programmatic trends, we modeled the odds of key program indicators (HIV serostatus and VIA positivity) with date of screening using restricted cubic splines, and presented them as probabilities for ease of interpretation. The analysis of VIA positivity is pooled across all years and assumes that the associations of the correlates of VIA positivity remain stable over time. R-software 2.11.1 (www.r-project.org ) was used for statistical analyses.

This outcomes evaluation study of anonymized patient records of a public sector implementation program was deemed exempt from human subjects review or the need for informed consent by the Research Ethics Committee of the University of Zambia.

## Results

### Screening Program Participant Characteristics by HIV Serostatus

**Figure 1:**
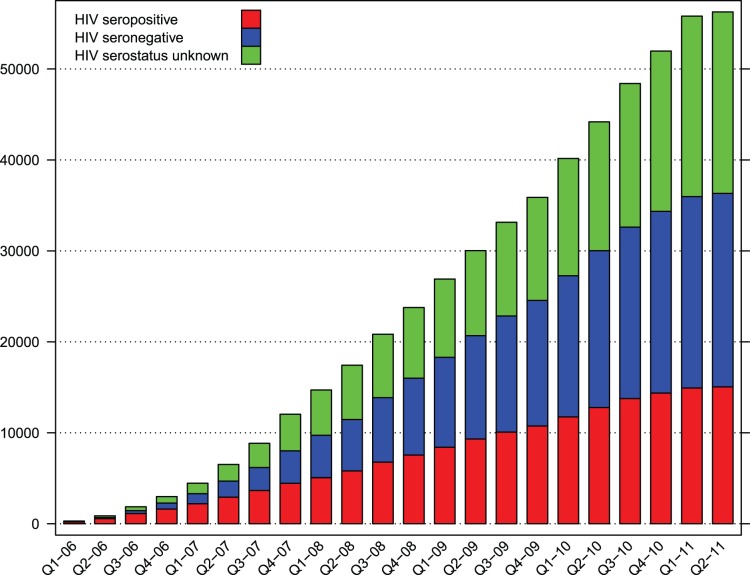
Cumulative enrollment of women in the Cervical Cancer Prevention Program in Zambia (CCPPZ) between January 2006–April 2011 by HIV serostatus.

CCPPZ provided screening services for a total of 56427 women in the period between January 2006 and April 2011 (date of data-freeze for current analysis). A total of 17 clinics were operated by 17 nurses and an outpatient surgery care center housing a Gynecologic Cancer Prevention Clinic. A total of 15081 (26.7%) women were HIV-seropositive, 21322 (37.8%) were HIV-seronegative, and 20024 (35.5%) reported not knowing their HIV serostatus at the time of enrollment (Table 1, Figure 1).

**Table 1:** Sociodemographic characteristics of the women utilizing services of the Cervical Cancer Prevention Program in Zambia (CCPPZ) between January 2006–April 2011 by HIV serostatus.

Characteristic		HIV-seropositive	HIV-seronegative	HIV serostatus unknown	Total	p-value*
		(n = 15081)	(n = 21322)	(n = 20024)	(n = 56427)	
**Age**	Median (IQR)	34 (29–40)	29 (24–37)	32 (26–40)	2 (26–39)	<0.001
	Missing data, n (%)	197 (1.3%)	234 (1.1%)	302 (1.5%)	733 (1.3%)	
**Education**, n (%)	Less than high school	4703 (46.3%)	6615 (39.3%)	6752 (44.3%)	18070 (42.8%)	<0.001
	High school completed	5464 (53.7%)	10225 (60.7%)	8480 (55.7%)	24169 (57.2%)	
	Missing	4914 (32.6%)	4482 (21.0%)	4792 (23.9%)	14188 (25.1%)	
**Marital status**, n (%)	Not Married	2882 (55.5%)	5847 (66.1%)	6306 (63.9%)	15035 (62.9%)	<0.001
	Married	2312 (44.5%)	3001 (33.9%)	3562 (36.1%)	8875 (37.1%)	
**Occupation**, n (%)	House wife	2805 (30.7%)	5917 (39.3%)	4838 (35.1%)	13560 (35.7%)	<0.001
	Formal sector	1982 (21.7%)	3301 (21.9%)	2723 (19.7%)	8006 (21.1%)	
	Informal sector	3226 (35.3%)	3659 (24.3%)	4387 (31.8%)	11272 (29.7%)	
	Other	1129 (12.3%)	2186 (14.5%)	1841 (13.4%)	5156 (13.6%)	
	Missing	5939 (39.4%)	6259 (29.4%)	6235 (31.1%)	18433 (32.7%)	
**Family income per month**, n (%)	Zambian kwacha ≤500,000	5666 (57.6%)	8501 (52.7%)	7565 (51.3%)	21732 (53.3%)	<0.001
	Zambian kwacha >500,000	4178 (42.4%)	7644 (47.3%)	7193 (48.7%)	19015 (46.7%)	
	Missing	5237 (34.7%)	5177 (24.3%)	5266 (26.3%)	15680 (27.8%)	
**Number of lifetime sexual partners**	Median (IQR)	3 (2–5)	2 (1–3)	2 (1–3)	2 (1–4)	<0.001
	Missing data, n (%)	263 (1.7%)	396 (1.9%)	902 (4.5%)	1561 (2.8%)	
**Number of pregnancies**	Median (IQR)	3 (2–5)	3 (2–5)	3 (2–5)	3 (2–5)	n.s.
	Missing data, n (%)	826 (5.5%)	2120 (9.9%)	2705 (13.5%)	5651 (10.0%)	
**Condom use with regular partner**, n (%)	Never	6855 (50.1%)	13104 (66.8%)	12383 (72.9%)	32342 (64.3%)	<0.001
	Ever	6830 (49.9%)	6523 (33.2%)	4608 (27.1%)	17961 (35.7%)	
	Missing data	1396 (9.3%)	1695 (7.9%)	3033 (15.1%)	6124 (10.9%)	

**Footnotes:** Abbreviation: IQR: interquartile range, HIV: human immunodeficiency virus, n.s.: not significant.

* p-value of comparison of characteristics between HIV-seropositive and HIV-seronegative women**.**

The median age of screened women was 32 years (interquartile range, IQR: 26–39). Over two-fifths (42.8%) of women with recorded educational status reported receiving less than a high school education. About a third (37.1%) of the screened population was married and living with spouses while the rest reported as being unmarried, widowed, or separated. Just over a fifth of the screened women (21.1%) reported being employed in the formal sector while over a third (35.7%) were housewives. A majority (53.3%) lived on a family income of less than 500,000 Zambian Kwacha per month (approximately equivalent to US$100). The median number of pregnancies reported was 3 (IQR: 2–5), the median number of lifetime sexual partners was 2 (IQR: 1–4) and just over a third (35.7%) reported ever use of a condom with a regular partner (Table 1).

HIV-seropositive women were more likely to be older than HIV-seronegative women (median age: 34 versus 29 years, p<0.001). Higher proportion of HIV-seropositive women were likely to be married (44.5% versus 33.9%, p<0.001), have less education (46.3% versus 39.3%, p<0.001), and have family incomes lower than 500,000 Zambian Kwacha per month (57.6% versus 52.7%, p<0.001) than HIV-seronegative women (Table 1). On average, HIV-seropositive women had a greater median number of lifetime sexual partners [3 versus 2 partners, p<0.001], while they reported higher rates of condom use with regular partners (49.9% versus 33.2%, p<0.001) than HIV-seronegative women (Table 1).

### Trends in Program Utilization by HIV-serologic Status

**Figure 2:**
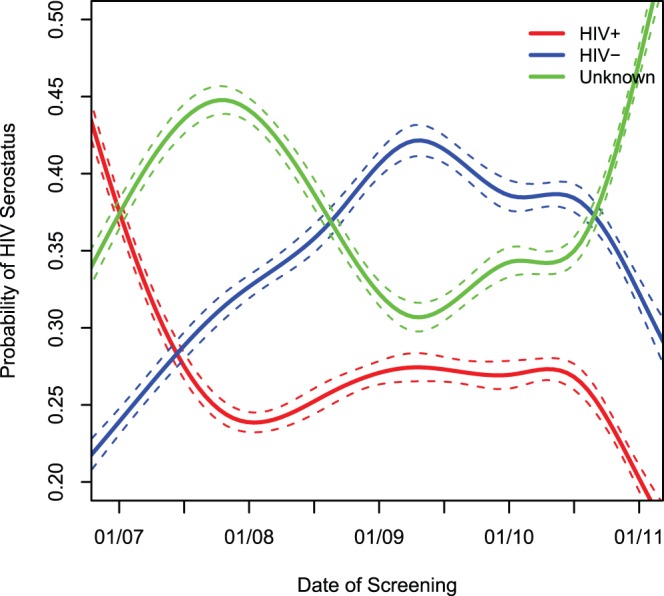
Probability of individual HIV serostatus among women utilizing services of the Cervical Cancer Prevention Program in Zambia (CCPPZ).

The relative proportion of women who were HIV-seropositive as a share of the total women who utilized the screening services declined over five years from 54% in 2006 to 23% in 2010. Consequently, the proportion of HIV-seronegative women increased in the same duration from 22% to 30%, while that of women with unknown HIV serostatus went up from 24% in 2006 to 39% in 2010. All these trends were statistically significant (p<0.001). The crude probability of the HIV serostatus of women utilizing the screening services is graphically depicted in Figure 2.

### Trends in Cervical Cancer Screening Results

**Figure 3:**
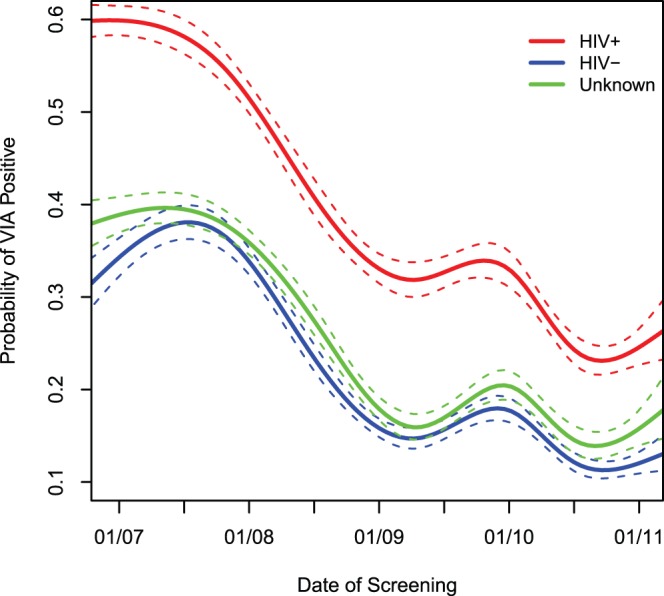
Probability of being detected as VIA positive stratified by HIV-serostatus among women utilizing services of the Cervical Cancer Prevention Program in Zambia (CCPPZ).

Cumulatively between January 2006 and April 2011, out of the total of 50,355 visual screening results available for analysis, 13,978 (27.7%) women were detected as VIA positive while 35,409 (70.3%) were VIA negative. Only a small minority (968, 1.9%) were classified as VIA-uncertain/indeterminate on initial screening. The VIA positivity rates declined over time from 47% in 2006 to 17% in 2010, and rates of VIA negativity increased from 52% in 2006 to 82% in 2010. This decline in VIA positivity, while directly coinciding with the increase in program scale-up activities, was consistent regardless of changes in utilization patterns by HIV serostatus, i.e., the crude probability of being detected as VIA positive (Figure 3, Y-axis) declined statistically significantly among women who were HIV-seropositive, HIV-seronegative, as well as those whose HIV serostatus was unknown (all p<0.001).

### Factors Associated with Cervical Cancer Screening Test Positivity

Overall, HIV-seropositive women had higher rates of VIA positivity (40.9%) than HIV-seronegative women (20.3%), while a quarter (25.2%) of women with unknown HIV serostatus were VIA positive. After covariate-adjustment in a multivariable logistic regression model, HIV-seropositive women had 2.62 times higher odds of being detected as VIA positive [adjusted odds ratio (AOR) 2.62 (95% CI: 2.49, 2.76, p<0.001)] than HIV-seronegative women (Table 2). Factors independently associated with VIA positivity included younger age, education beyond high school, having income under 500,000 Zambian Kwacha per month, reporting two or more lifetime sexual partners, reporting lower age of sexual debut and reporting ever using condoms with regular partner (Table 2).

**Table 2:** Multivariable logistic regression model to identify factors associated with VIA screening test positivity among women utilizing services of the Cervical Cancer Prevention Program in Zambia (CCPPZ) between January 2006–April 2011.

		Risk of VIA positivity	
		Adjusted Odds ratio (95% CI)	p-value
**Age**	≤25 years	1 (Referent)	<0.001
	30 years	1.00 (0.97, 1.03)	
	35 years	0.87 (0.82, 0.91)	
	≥40 years	0.74 (0.69, 0.79)	
**Education**	Less than high school	1 (Referent)	<0.001
	High school completed	0.89 (0.85, 0.94)	
**Marital Status**	Not married	1 (Referent)	0.155
	Married	0.95 (0.88, 1.02)	
**Occupation**	House wife	1 (Referent)	0.930
	Formal sector	1.00 (0.91, 1.09)	
	Informal sector	1.01 (0.93, 1.08)	
	Other	0.98 (0.90, 1.07)	
**Family income per month**	Zambian kwacha ≤500,000	1 (Referent)	0.003
	Zambian kwacha >500,000	0.92 (0.87, 0.97)	
**Number of lifetime sexual partners**	1 partner	1 (Referent)	<0.001
	2 partners	1.10 (1.04, 1.16)	
	3 partners	1.16 (1.10, 1.23)	
	4 partners	1.20 (1.13, 1.27)	
**Age of sexual debut**	(per year increase)	0.98 (0.98, 0.99)	<0.001
**Number of pregnancies**	1	1 (Referent)	<0.001
	3	1.16 (1.09, 1.23)	
	5+	1.02 (0.95, 1.10)	
**Condom use with regular partner**	Never	1 (Referent)	<0.001
	Ever	1.17 (1.11, 1.22)	
**HIV serostatus**	HIV-seronegative	1 (Referent)	<0.001
	HIV-seropositive	2.62 (2.49, 2.76)	
	HIV serostatus unknown	1.41 (1.34, 1.48)	

**Footnotes:** Abbreviations: VIA: visual inspection with acetic acid, HIV: human immunodeficiency virus.

Following a *post hoc* adjustment for date of screening to account for potential confounding effect of calendar time, the covariate associations are unchanged in direction of effect size as well as tests of significance.

## Discussion

VIA-based screening is increasingly being adopted for cervical cancer prevention interventions in the developing world. CCPPZ is the first and largest PEPFAR-supported VIA-cryotherapy based ‘screen-and-treat’ cervical cancer prevention initiative linked to HIV/AIDS care and support programs in the developing world [10], [19]–[21], [24]. Developed initially as a program targeting HIV-infected women (who had very high cervical disease burden [25], [26]); CCPPZ now screens a vast majority of women in the implementation catchment areas who are not HIV-seropositive. In this paper, we have provided the world’s first real-world implementation-based evidence that even while focused on the highest-risk HIV-infected women, utilization of ‘screen-and-treat’ programs by the broader general population occurs with sustained efforts.

HIV/AIDS care and treatment programs provide very highly suitable platforms for launching ‘screen-and-treat’ cervical cancer prevention services, a very critical and life-saving intervention for all eligible women, regardless of their HIV serostatus. As we have demonstrated through this study, the increasing utilization of program services by women other than who are HIV-seropositive has coincided with program expansion in response to increased demand, and greater acceptance and uptake of screening services in the general population. Sustainability of the screening program has been achieved through community involvement and continued training of non-physician community mobilizers and care givers [27]. Scale-up of cervical cancer prevention interventions in this high-HIV prevalence setting is being accompanied by efforts for dispelling misconceptions that only “people with AIDS” could suffer from cancer of the cervix, attempts for de-stigmatization of cervical cancer in general, and improving the awareness and importance of screening in particular [28]. Nevertheless, given their increased risk for cervical cancer, it is important to continue targeted screening efforts for HIV-seropositive women, particularly linked to their cART clinic visits. The increased utilization of screening services by women who have never tested for HIV, and those who have previously (>6 months ago) tested seronegative, also gave an opportunity to offer simultaneous counseling and serological testing for HIV in the cervical cancer clinics for such women. This activity has allowed linkages to care of women who newly test HIV-seropositive, thereby achieving even greater programmatic efficiencies (CCPPZ, unpublished data).

The successful scale up of cervical cancer screening activities was aided by expansion from the initial 2 clinics in 2006 to 17 clinics by 2011 and from 5 nurses in 2006 to 17 nurses in 2011. CCPPZ program nurses form the backbone of the ‘screen-and-treat’-based program operations. They function as the primary care givers independently performing screening and same-visit cryotherapy procedures, and refer patients needing histologic evaluation to tertiary care facilities. The training of nurses has been a vital component, largely achieved through a train-the-trainer model, where nurses serve as educators for their peers. This model has also proven successful in training nurses from 12 other African countries who have received on-site clinically-mentored training within CCPPZ-supported government clinics as part of a global cervical cancer training program.

One of the most important factors in the improvement of VIA performance is the use of digital camera enhancement of the cervix (‘digital cervicography’), a low-cost adaptation for the Zambian setting. This is modeled after colposcopy, yet does not require a costly colposcope or prolonged training [19], [29]–[31]. It also has substantial collateral benefits, including the ability to project the cervical image on the camera monitor or a bedside television/computer screen for patients to visualize simultaneously (thereby achieving patient education), electronic transmission of the image by email or cellphone to a consulting gynecologist for telemedicine support for decision making (if needed), and the ability to routinely undertake quality assurance on the images [19]. These attributes have allowed for overall reductions in the misclassifications of VIA, as reflected by the decreasing rates of VIA positivity over the 2006–2011 calendar years period. As a rater-dependent screening method, VIA inherently suffers from the same challenges as other visual interpretation methods including colposcopy and cytology [16]–[18]. The greater the experience and number of procedures performed correctly by the provider, the less the chances of errors. Indeed, one of the most common criticisms of VIA is the tendency to ‘overcall’ an acetowhite appearing cervix as ‘VIA positive’. However, as we have shown in this study, with the increase in the experience of nurses undertaking screening (as reflected by the total number of women utilizing the screening services), the rate of VIA positivity has fallen substantially, regardless of the HIV serostatus. Since the eventual successes in reduction of cervical cancer burden will be dependent on how well are screening services implemented in a particular setting, this unique demonstration through program operations data underscores the importance of a sustained focus on evaluation, training and quality improvement in screening implementation.

In the wake of projections of a startling rise in new cancer cases and deaths in Africa over the next 20 years [32], [33], our experience in initiating and expanding cervical cancer ‘screen-and-treat’ services in Zambia may provide useful insights for African governments and their international partners as they consider implementing cancer prevention initiatives, especially in high HIV prevalence settings, to mitigate this impending tragedy.
